# Time series model for forecasting the number of new admission inpatients

**DOI:** 10.1186/s12911-018-0616-8

**Published:** 2018-06-15

**Authors:** Lingling Zhou, Ping Zhao, Dongdong Wu, Cheng Cheng, Hao Huang

**Affiliations:** Department of Information, Research Institute of Field Surgery, Daping Hospital of Army Medical University, 10 Changjiang Access Road, Chongqing, 400042 China

**Keywords:** New admission inpatients, Time series forecasting, SARIMA model, NARNN model, Hybrid model

## Abstract

**Background:**

Hospital crowding is a rising problem, effective predicting and detecting managment can helpful to reduce crowding. Our team has successfully proposed a hybrid model combining both the autoregressive integrated moving average (ARIMA) and the nonlinear autoregressive neural network (NARNN) models in the schistosomiasis and hand, foot, and mouth disease forecasting study. In this paper, our aim is to explore the application of the hybrid ARIMA-NARNN model to track the trends of the new admission inpatients, which provides a methodological basis for reducing crowding.

**Methods:**

We used the single seasonal ARIMA (SARIMA), NARNN and the hybrid SARIMA-NARNN model to fit and forecast the monthly and daily number of new admission inpatients. The root mean square error (RMSE), mean absolute error (MAE) and mean absolute percentage error (MAPE) were used to compare the forecasting performance among the three models. The modeling time range of monthly data included was from January 2010 to June 2016, July to October 2016 as the corresponding testing data set. The daily modeling data set was from January 4 to September 4, 2016, while the testing time range included was from September 5 to October 2, 2016.

**Results:**

For the monthly data, the modeling RMSE and the testing RMSE, MAE and MAPE of SARIMA-NARNN model were less than those obtained from the single SARIMA or NARNN model, but the MAE and MAPE of modeling performance of SARIMA-NARNN model did not improve. For the daily data, all RMSE, MAE and MAPE of NARNN model were the lowest both in modeling stage and testing stage.

**Conclusions:**

Hybrid model does not necessarily outperform its constituents’ performances. It is worth attempting to explore the reliable model to forecast the number of new admission inpatients from different data.

**Electronic supplementary material:**

The online version of this article (10.1186/s12911-018-0616-8) contains supplementary material, which is available to authorized users.

## Background

With an increasing global population and economy, the demand for healthcare continues to rise. Hospital crowding has become a major problem faced by large hospitals. Hospital adverse events increase with crowding, and have further effects on patient satisfaction, quality of nursing, treatment, wait time, and length of stay [1–4]. A vast literature about overcrowding focus on the outpatient wards [1, 5] and emergence departments [4, 6]. Overcrowding appearing in the inpatient wards should also be paid attention to. When no inpatient beds are available to admit new inpatients, overcrowding would occur. Often, inpatient beds may be scarce as a result of too many patients with non-urgent medical conditions seeking healthcare.

The prediction of admissions is one piece of larger equation in the using hospital census, patient acuity, disease burden, allocation of resources and general management to improve hospital performance and improve patient outcomes. Much of research on hospital management focuses on the emergence of demand predicting [7–10], forecasting of outpatient visits [11, 12], inpatients discharge [13], and patient volume [14]. However, little published research is available regarding predicting the number of new admission inpatients. Monitoring and forecasting for new admission inpatients are important processes in making feasibility decisions for hospital resource management, reducing crowding, and improving the quality of medical care delivered.

Time series forecasting approaches have been adopted in other research fields, such as infectious disease [15–18], power and energy [19], finance and economy [20, 21], traffic [22], environment [23], and hydrology [24]. Among these approaches, for problems involving linear time series forecasting, the autoregressive integrated moving average (ARIMA) model is linear in that predictions of the future values are constrained to be linear functions of past observations. However, the prediction accuracy of ARIMA model is restricted due to its inability to capture the nonlinear relationships of time series in the real world. For nonlinear problems, the artificial neural network (ANN) has enhanced forecasting accuracy due to its intrinsic properties that can approximate any sort of arbitrary nonlinear function [25]. More recently, hybrid forecasting models that combine the ARIMA and ANN models to handle linear and nonlinear relationships that exist in time series data have been extensively applied in many fields with high predictive performance [16, 17, 19, 21, 26–28] . These previous studies remind us that the number of new admission inpatients as time series could also be predicted by hybrid models.

Our team has successfully applied the hybrid model with ARIMA and the nonlinear autoregressive neural network (NARNN) to the field of infectious diseases, for example forecasting the prevalence of schistosomiasis in humans in Qianjiang City and Yangxin City, China [17, 28], and the incident cases of hand, foot, and mouth disease in Shenzhen, China [29]. Wu [16] also verified the feasibility of a hybrid ARIMA-NARNN model in forecasting the incidence of hemorrhagic fever with renal syndrome in Jiangsu Province, China. These literatures indicate combining both the ARIMA and NARNN models could improve the forecasting performance due to incorporate both the linear and nonlinear patterns found in the real world.

In this paper, we will explore whether the ARIMA-NARNN hybrid model is reliable for forecasting the number of new admission inpatients to a large hospital. Our aim is to forecast the monthly and daily new admission inpatients using time series models. This will enable hospitals to provide more efficient and better quality care to their patients.

## Methods

### Data sources

Our hospital, as a member of the first batch of public tertiary hospitals in Chongqing, China, is a large-scale comprehensive medical institution involves in medical care, education and scientific research. By now our hospital opens with 2628 inpatient beds, and there are almost 2,000,000 outpatients, 100,000 emergency admissions and 100,000 discharges during a year. Like most other tertiary hospitals in China, we are faced with the growing challenge of overcrowding. Between 2010 and 2015, the amount of outpatient-emergency patients, new admissions and surgeries increased by 96.75, 37.59, 37.13%, respectively. Although the largest increase was observed in the number of outpatient-emergency patients, allocation of hospital resourcesis also greatly effected by admitted patients. Therefore, we chose to focus on new admissions in this study.

To analyze the “day of the week” effect and the “month of the year” effect of new admission inpatients, we included data from two different time series: monthly data from January 2010 to October 2016 (82 months) and daily data from January 4 to October 2, 2016 (273 days). The data was obtained from the Hospital Information System (Additional file 1). The study was approved by the ethics committee of Daping Hospital of Third Military Medical University.

### Methods

#### The SARIMA model construction

Taking into account the characteristics of seasonal fluctuation of new admission inpatients, the seasonal ARIMA (SARIMA) model was constructed. The SARIMA (*p*, *d*, *q*)(*P*, *D*, *Q*)*s* model is developed from the ARIMA model [15]. There are seven main parameters in the SARIMA model: the order of autoregressive (*p*) and seasonal autoregressive (*P*), the order of regular difference (*d*) and seasonal difference (*D*), and the order of moving average (*q*) and seasonal moving average (*Q*), and finally, the length of seasonal period(*s*). Stationarity is a necessary condition in building a SARIMA model and differencing is often used to stabilize the time series data. The main methods to check the stationarity of time series include the sequence trend diagram, autocorrelation function (ACF), partial autocorrelation function (PACF), augmented dickey-fuller (ADF) unit root test, phillips and perron (PP) test, nonparametric test and so on. In this study, the ACF, PACF plots, and ADF test were used to identify the stationarity of time series and the possible order of autoregression and moving average. The most suitable model was selected according to the akaike information criterion (AIC), schwarz bayesian criterion (SBC) and the Ljung-Box Q-test. Both monthly and daily seasonal periodicities were taken into account in this analysis. The two time series are nonstationary. Regular difference and seasonal difference are used to stabilize them. The new stationary series after difference are as the target sequence of the SARIMA model.

Before the modeling process, the time series were split into two sets each: one (modeling data set) was used to develop the models and the other (testing data set) to test the model. The modeling monthly set included data from January 2010 to June 2016 (1/2010–6/2016), while data from July to October 2016 (7/2016–10/2016) was used as the corresponding testing data set. The modeling daily data set was from January 4 to September 4, 2016 (1/4/2016–9/4/2016), while the testing data was collected within 1 week from September 5 to September 11, 2016(9/5/2016–9/11/2016) and four-weeks from September 5 to October 2, 2016 (9/5/2016–10/2/2016). The SARIMA model was developed with SAS Software version 9.4.

#### The NARNN model construction

**Fig. 1:**
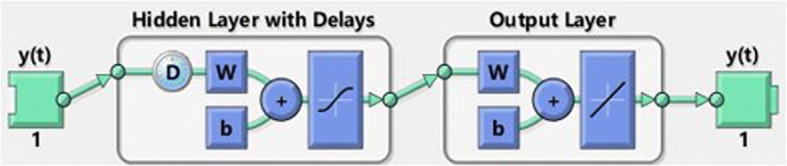
The configuration of the NARNN. The NARNN consists of one output layer with 1 unit and one hidden layer with n units and D delays

The NARNN model is capable of predicting a simple time series given past values of the same time series, *y*_*t*_ = *f*(*y*_*t* − 1_, *y*_*t* − 2_, ⋯, *y*_*t* − *d*_). NARNN incorporates a default two-layer FFBP with a sigmoid transfer function in the hidden layer, a linear transfer function in the output layer. The output of the NARNN, *y*(*t*), is fed back to the input of the network (through delays). The configuration is showed in Fig. 1. The NARNN model was performed with the Neural Network Toolbox in MATLAB version 7.11(R2010b). The following steps describe how to build the NARNN model.

- Step 1: Inputted the target series to generate a command-line script.
- Step 2: Used the default data division function type to divide the data randomly to three parts: the training subset (training the network), the validation subset (stopping training before over-fitting) and the testing subset (testing the network generalization). Set the ratios for training (80%), validation (10%), and testing (10%).
- Step 3: Adjusted the arguments feedback delays and hidden units by trial and error. Set the hidden units (10~ 18) and feedback delays (4~ 10) depending on our experience with the amount of data. In total of 63 architectures were tested to obtain the optimal model according to the error autocorrelation plot, the time series response plot, the MSE and the correlation coefficient (R).
- Step 4: According to the feedback delays, we inputted the targets of the closed loop network for multi-step-ahead prediction.

#### The hybrid SARIMA-NARNN model construction

The hybrid SARIMA-NARNN model was developed in two stages. In the SARIMA model stage, the main goal was to extract the linear relationships between the original data. The SARIMA model was then used to generate the residuals. In the NARNN model stage, the chief aim was to model the nonlinear relationships that exist in the residuals. The eventual combined forecasting values of the time series were the sum of predictions from SARIMA model and adjusted residuals from NARNN model: ${\widehat{y}}_t={\widehat{L}}_t+{\widehat{N}}_T$, where ${\widehat{y}}_t$ was the predicted value by the SARIMA-NARNN model at time t, ${\widehat{L}}_t$ denoted the predicted value by the SARIMA model at time t, and ${\widehat{N}}_t$ denoted the residuals predicted by the NARNN model.

### Performance statistic index

The modeling errors and testing errors were used to compare the fitness and prediction performance of the SARIMA, NARNN and SARIMA-NARNN models. The three indices: root mean square error (RMSE), mean absolute error (MAE) and mean absolute percentage error (MAPE), were selected for evaluation of the errors. The formulas for calculation are defined as follows:

1 $$ RMSE=\sqrt{\frac{1}{n}\sum \limits_{t=1}^n{\left({y}_t-\overset{\wedge }{y_t}\right)}^2} $$

2 $$ MAE=\frac{1}{n}\sum \limits_{t=1}^n\left|{y}_t-\overset{\wedge }{y_t}\right| $$

3 $$ MAPE=\frac{1}{n}\sum \limits_{t=1}^n\frac{\left|{y}_t-\overset{\wedge }{y_t}\right|}{y_t} $$

## Results

### SARIMA model analysis

**Fig. 2:**
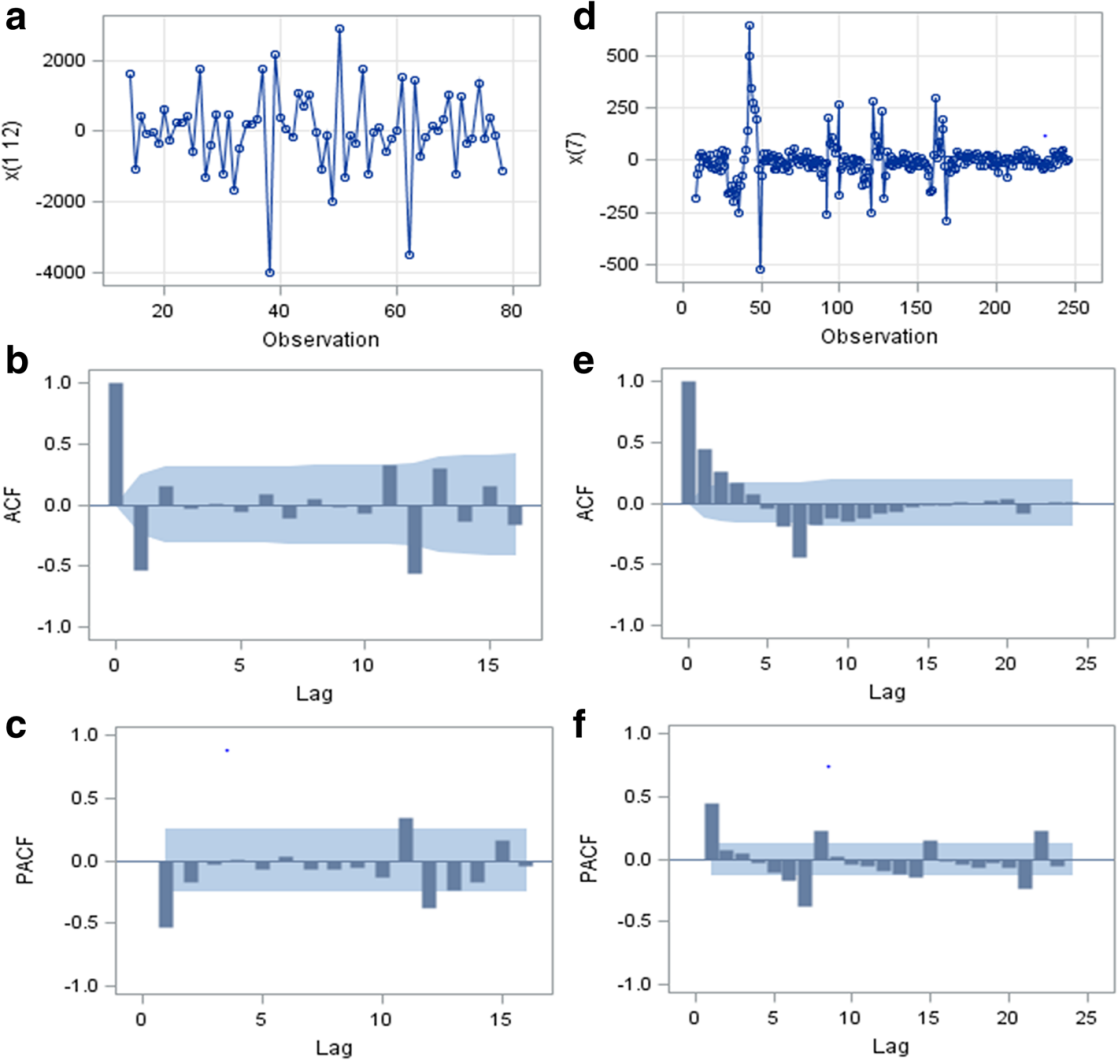
Trend and Correlation Analysis for different time series. **a**, **b** and **c** show the trend of new admission inpatients per month from January 2010 to June 2016, ACF and PACF plots of monthly original time series (MOS) respectively after one order of regular difference and one order of seasonal difference with the length of seasonal period 12. **d**, **e** and **f** show the trend of new admission inpatients per day from January 4 to September 4, 2016, ACF and PACF plots of daily original time series (DOS) respectively after one order of seasonal difference with the length of seasonal period 7

The monthly time series achieved stationary state after regular difference of 1 order, followed by seasonal difference of 1 order and length of seasonal period of 12. The daily time series achieved stationary state after seasonal difference of 1order and length of seasonal period of 7 without regular difference. Fig. 2 a and d show the stationary monthly original time series (MOS) and daily original time series (DOS) after difference. The ACF and PACF plots of MOS and DOS after difference are displayed in Fig. 2 b, c, e, and f. Most of the correlations were at around zero within a 95% confidence interval, suggesting that the time series achieved stationarity. Results of ADF test of MOS and DOS after difference was considered are shown in Table 1. All the *P-*values were less than 0.05 supporting the absence of unit root. This provided further confirmation that the difference in the series was stationary.

**Table 1:** Augmented dickey-fuller unit root (ADF) test of two time series

Type	Lag	Monthly		Daily	
		*t*	*P*	*t*	*P*
Zero Mean	0	−14.60	< 0.0001	−9.56	<.0001
	1	−8.15	< 0.0001	−7.55	<.0001
Single Mean	0	−14.48	0.0001	−9.54	<.0001
	1	−8.09	0.0001	−7.54	<.0001
Trend	0	−14.37	< 0.0001	−9.52	<.0001
	1	−8.02	< 0.0001	−7.52	<.0001

Note: Monthly = monthly time series from January 2010 to June 2016

Daily = daily time series from January 4 to September 4,2016

Results of the parameter estimation are shown in Table 2. All of the estimated parameter values were statistically significant (*P* < 0.05). These results showed that using the model SARIMA(1,1,0)(0,1,1)_12_ with the smallest AIC (1049.72) and SBC (1054.07) for forecasting the monthly new admission inpatients and the model SARIMA(2,0,1)(0,1,1)_7_ (AIC = 1049.72, SBC = 1054.07) for daily predicting were appropriate.

**Table 2:** Parameter estimations of two time series from SARIMA model

Time series	Parameter	Estimate	Standard error	*t*	*P*	Lag
Monthly	MA1,1	0.90	0.08	11.54	<.0001	12
	AR1,1	−0.50	0.11	−4.52	<.0001	1
Daily	MA1,1	−0.45	0.06	−7.05	<.0001	1
	MA2,1	0.81	0.04	21.32	<.0001	7
	AR1,1	0.23	0.07	3.34	0.0010	2

The autocorrelation of residuals is presented in Table 3. All the *P*-values were more than 0.05, showing that the residuals were all white noises, which indicated the information was extracted sufficiently.

**Table 3:** White noise check of residuals of two time series from SARIMA model

Lag	Monthly		Daily	
	*Χ* ^*2*^	*P*	*Χ* ^*2*^	*P*
6	4.35	0.36	3.06	0.38
12	7.20	0.71	6.45	0.69
18	11.04	0.81	9.80	0.83
24	13.52	0.92	13.14	0.90

All predicted values are available in the Additional file 2. We then computed the monthly residual series (MRS) and daily residual series (DRS), which were subsequently applied as the target series of the NARNN model.

### NARNN model analysis

The optimal NARNN models we applied to forecast the MOS, MRS, DOS and DRS are shown in Table 4: target series MOS with hidden units 11 and delays 8, MRS with hidden units 16 and delays 6, DOS with hidden units 13 and delays 10, and DRS with hidden units 14 and delays 7. All MSE of the training, validation, and testing subsets were relatively small, and all the R values were greater than 0.8.

**Table 4:** Optimum network parameters of different target series

Time series	Target series	Hidden units	Delays	RMSE			*R*
				training	validation	testing	
Monthly	OS	11	8	53.74	309.8	280.89	0.92
	RS	16	6	113.58	187.02	221.44	0.82
Daily	OS	13	10	30.07	36.01	46.48	0.96
	RS	14	7	33.91	51.35	41.90	0.87

Note: OS = original series, RS = residual series

**Fig. 3:**
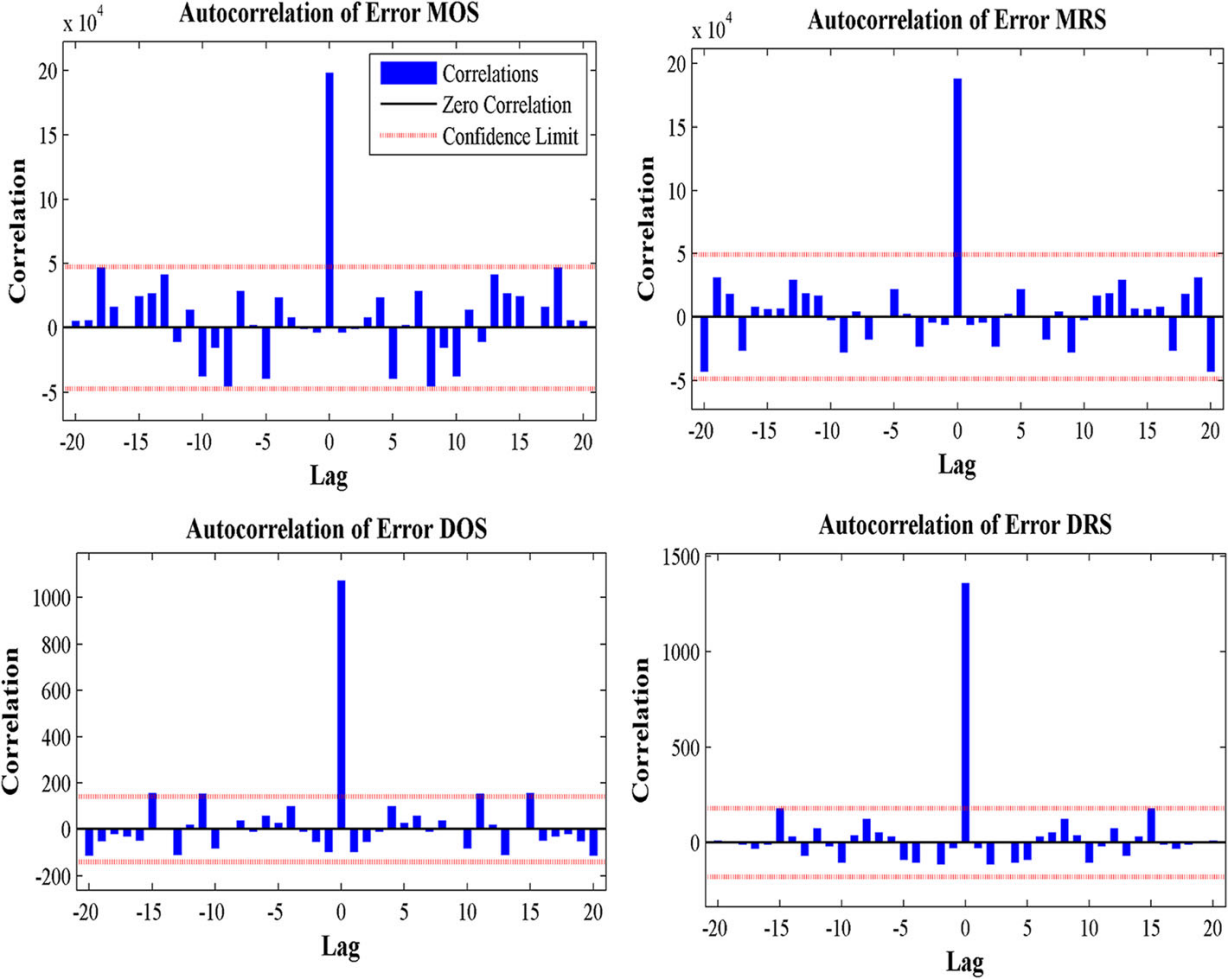
Error autocorrelation plots of different time series from NARNN model. The error autocorrelation was one of the evaluation indices in the modeling process. The red dotted line indicate 95% confidence intervals. MOS = monthly original time series, MRS = monthly residual series, DOS = daily original time series, DRS = daily residual series

**Fig. 4:**
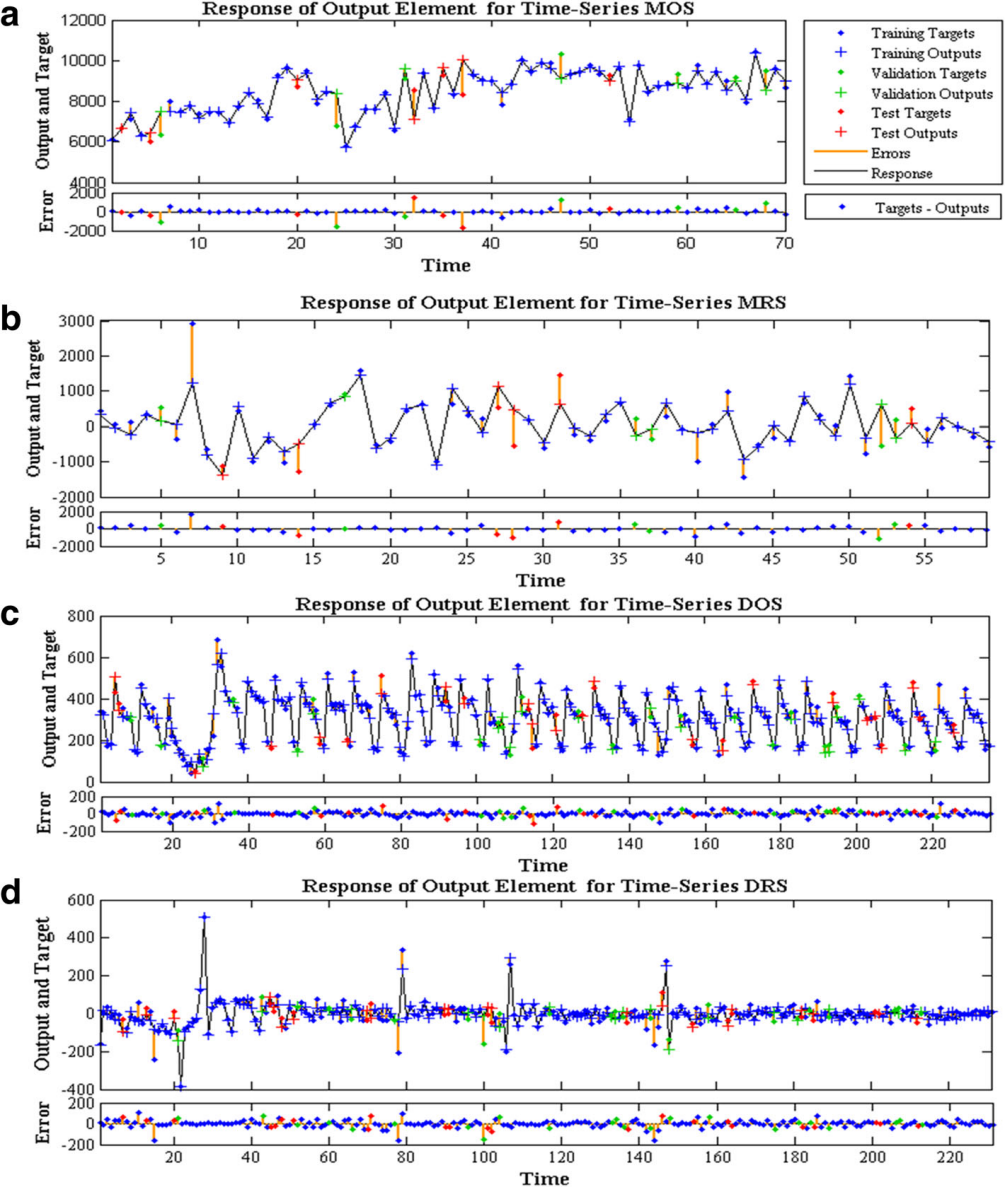
The time-series response plots of different time series from NARNN model. **a**, **b**, **c** and **d** show the inputs, targets, and errors versus time and also give which time points were selected for training, testing, and validation

The error autocorrelation function plot of different target series are displayed in Fig. 3. The correlation coefficients for all the models, except for the one at zero lag, fell within the 95% confidence limits, demonstrating that the models were applicable. The time series response plots are displayed in Fig. 4, showing that the outputs were distributed evenly on both sides of the response curve and the errors were small in the training, testing, and validation subsets, indicating that the model reliably reflected the data. We observed that the predicted residuals from July to October 2016 were − 240.47, 35.31, − 132.87 and 189.98, respectively. In addition, the predicted residuals, from September 5 to October 2, 2016 were 3.86, 3.65, 7.93, 6.17, 5.50, 5.46, 10.44, 10.65, 11.41, 14.96, 14.14, 17.08, 18.08, 21.26, 23.60, 24.68, 29.83, 29.77, 35.42, 37.97, 41.36, 48.66, 47.95, 60.84, 58.58, 71.15, 79.63 and 71.89 respectively. The predicted monthly and daily new admission inpatients by NARNN model are presented in the Additional file 2.

### SARIMA-NARNN model analysis

**Fig. 5:**
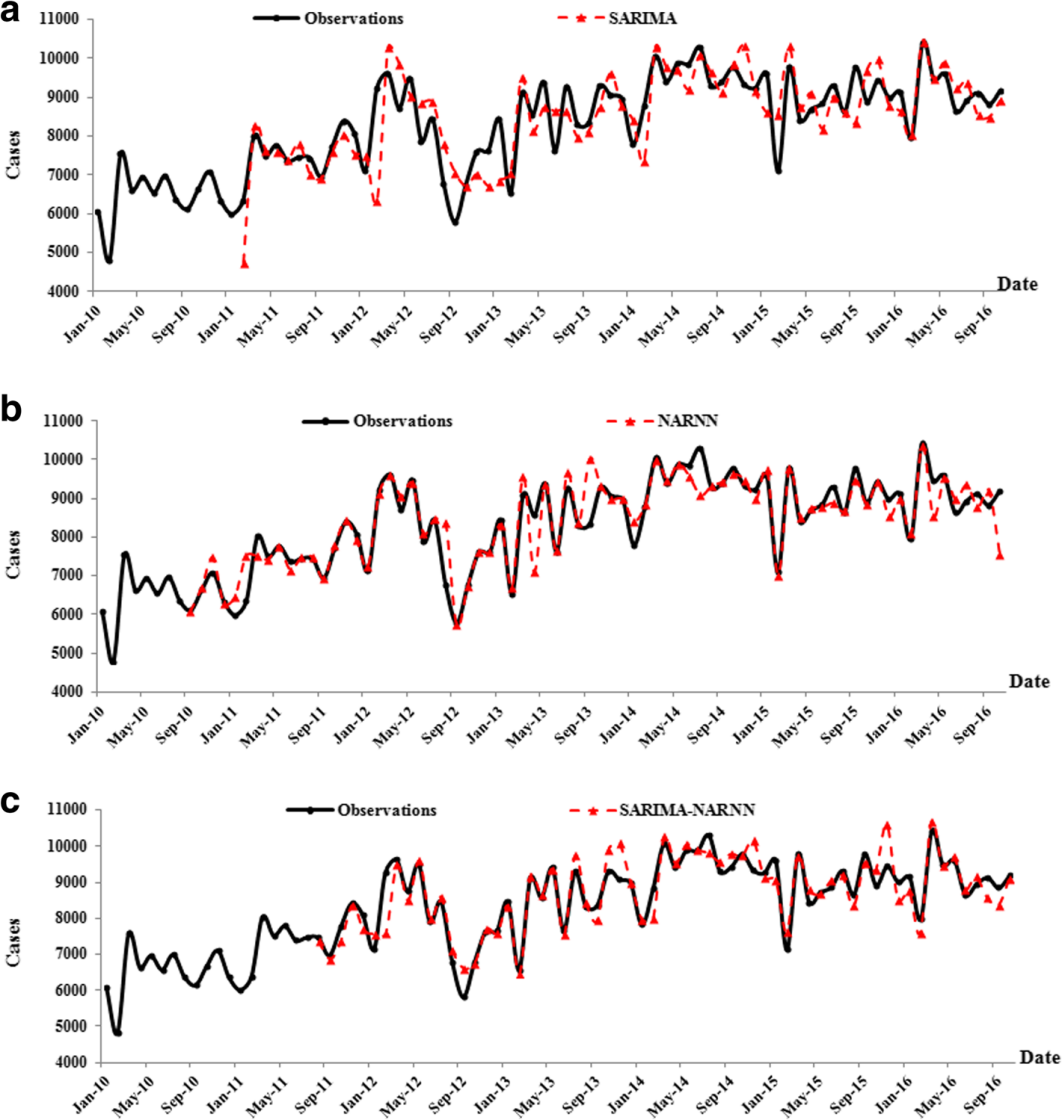
The change trend of the monthly number of new admission inpatients from three models. **a**, **b** and **c** show the observations and predicted values from the SARIMA model , NARNN model and SARIMA-NARNN model respectively

**Fig. 6:**
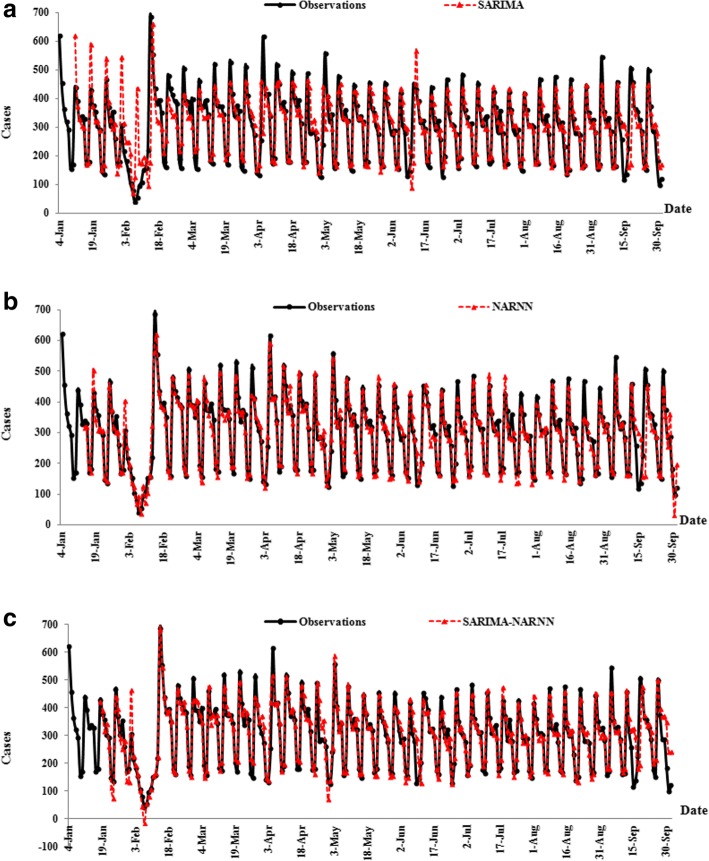
The change trend of the daily number of new admission inpatients from three models. **a**, **b** and **c** show the observations and predicted values from the SARIMA model , NARNN model and SARIMA-NARNN model respectively

The monthly and daily values predicted by the SARIMA-NARNN model are shown in the Additional file 2. The point-to-point comparison between original observations and predicted values from the SARIMA, NARNN and SARIMA-NARNN models are shown in Fig. 5 and Fig. 6. The curve of the original observations and predicted series from the SARIMA-NARNN model was closer than those from the SARIMA and NARNN models (Fig. 5 a, b and c), indicating that the hybrid model was well fitted to the data of monthly new admission inpatients. However, among the three models, the predicted curve from the NARNN model was the closest to the original curve (Fig. 6 a, b and c), indicating that the NARNN model was appropriate for forecasting the daily new admission inpatients.

### Comparing analysis

The differences in modeling errors and testing errors between the original observations and predicted values of monthly and daily new admission inpatients are presented in Table 5.

**Table 5:** Prediction performance results of three models

Time	Model	Modeling error			Testing error		
series		RMSE	MAE	MAPE*	RMSE	MAE	MAPE*
Monthly	SARIMA	759.67	573.75	6.81	426.45	410.97	4.56
	NARNN	448.04	232.92	2.73	886.39	692.29	7.61
	SARIMA-NARNN	433.82	299.22	3.48	378.03	327.75	3.64
Daily	SARIMA	73.73	42.01	18.50	37.29 ^a^	20.36 ^a^	4.91 ^a^
					87.01^b^	51.11 ^b^	24.22 ^b^
	NARNN	32.97	23.49	9.42	24.54 ^a^	14.12 ^a^	3.44 ^a^
					86.88 ^b^	49.24 ^b^	23.14 ^b^
	SARIMA-NARNN	36.86	25.59	10.37	35.82 ^a^	18.76 ^a^	5.15 ^a^
					98.24 ^b^	63.43 ^b^	33.90 ^b^

*MAPE values should be multiplied by 10^−2^. ^a^the testing error in one-week, ^b^the testing error in four-weeks

For the monthly data, the modeling RMSE and the testing RMSE, MAE and MAPE of the SARIMA-NARNN model were less than those obtained from the single SARIMA or NARNN model, but the MAE and MAPE were more than those obtained from NARNN model.

For the daily data, we calculated the testing errors of one-week and four-weeks. The NARNN model was the best with the lowest RMSE, MAE and MAPE in modeling stage and testing stage, indicating that the NARNN model was well fitted to the data of daily new admission inpatients.

## Discussion

To our knowledge, this study was the first to develop and apply the time series models in admission patients research, with the specific purpose of forecasting the number of new admission inpatients trends and guiding management strategies. We sought to construct a single SARIMA model, a single NARNN model, and a hybrid SARIMA-NARNN model based on the monthly and daily data of an entire hospital. The NARNN model and SARIMA-NARNN model were appropriate to forecast the number of new admission inpatients. But the results of forecasting performance were compared by using the RMSE, MAE, MAPE showing that the hybrid model does not necessarily achieved better prediction accuracy than either of the models used separately.

As shown in Fig. 5, the original new admission inpatients fluctuated every year based on the monthly data. However, an upward trend was observed overall. The result of the SARIMA model analysis incorporated a 12-step seasonal differencing operation. The monthly time series analysis supports a “month of the year” effect. The lowest numbers were observed in January or February each year, presumably due to the Spring Festival holiday. The numbers reached the maximum in March 2010, 2012, 2015 and 2016, and greater numbers in March compared to other months were also observed in other years, a phenomenon that could potentially be attributed to long holiday and seasonal replacement. Based on these findings, we suggest that hospital management should strategize and assign medical resources accordingly. The modeling RMSE, MAE, MAPE of the SARIMA-NARNN model decreased by 42.89, 47.85, 48.86% and the corresponding testing error decreased by 11.35, 20.25, 19.99%, respectively as compared to using the SARIMA model alone. When compared to the NARNN model, the modeling RMSE of the SARIMA-NARNN model decreased by 3.12%, and the testing RMSE, MAE, MAPE decreased by 57.35, 52.66, 52.11%, respectively. Interestingly, the modeling MAE and MAPE of the SARIMA-NARNN model increased by 28.47 and 27.26%, respectively. As mentioned in the article [30, 31], the RMSE is not always a superior parameter over the MAE, a combination of metrics is often required to accurately evaluate model performance. However, all testing errors of the SARIMA-NARNN model were the lowest among the three models and overall, the predicted curves of the hybrid model was close to the original curves (Fig. 5 a, b and c). Therefore, we concluded that the hybrid model was the most appropriate for forecasting the monthly new admission inpatients.

As shown in Fig. 6a, b and c, our analysis of daily data indicates an obvious “day of the week” effect. Maximum values were usually observed on Mondays, while the minimum values tended to fall on Saturdays or Sundays every week. Some fluctuations were found under the influence of various festivals. For examples, the lowest number was observed during the 7th to the 13th of February likely due to the Spring Festival holiday and the one-week maximum was observed on Tuesday (3th of May) probably because this was the first day after the May Day holiday. In addition, the maximum value was also found on Sunday (18th of September) potentially due to the Mid Autumn Festival holidays from Thursday to Saturday prior. Forecasting performance could be greatly influenced by these fluctuations. If the time series predictions were within the range of these holidays, extra cautions should be paid on interpreting prediction results. As compared to using the SARIMA model alone, the modeling RMSE, MAE, and MAPE of the NARNN model decreased by 55.28, 44.01, and 49.01% and the corresponding one-week and four-weeks testing errors dropped by 34.20, 30.65, 30.05 and 0.15%, 3.66, and 4.45%, respectively. When compared to the SARIMA-NARNN model, the modeling RMSE, MAE, MAPE of the NARNN model decreased by 10.54, 8.22 and 9.23%, respectively, while the corresponding one-week and four-weeks testing errors reduced by 31.50, 24.74, 33.33 and 11.56%, 22.37, 31.72%, respectively. We, therefore, concluded that the NARNN model was suitable for forecasting the daily new admission inpatients.

According to the development trend of new admission inpatients, we can make some following suggestions for the hospital managers. Try to avoid the medical staff leave at the peak of admission; Carry out the repair work for the inpatient beds on Saturday or Sunday; Provide vacant beds by clinical departments with fewer admission inpatients to other departments with more admission inpatients. Set up some waiting beds for turnover in the whole hospital; Make an “emergence plan about overcrowding”- once overcrowding occur the “overcrowding beds” are opened. When the forecasting results indicate that the new admission inpatients are increasing, the plan is in a state of vigilance.

Although the ARIMA model is one of the most mature time series forecasting methods, our study [17, 28] and other studies [32] have indicated that its forecasting performance for predicting real world cases is slightly lower than other models. Therefore, we do not recommend using the ARIMA model exclusively. The NARNN model is capable of successfully simulating some time series due to its dynamic property, high fault tolerance performance, and ability to capture nonlinear information [25, 33]. In practical data analysis, the NARNN model should be construct. In addition, our results were consistent with previous publication, which reported the comparative study of autoregressive neural network hybrids, showing that hybrid models are not always better and the model construction process should remain an important step despite the popularity of hybrid models [34]. The four-weeks testing errors were much greater than those of one-week, showing that the prediction accuracy was obviously reduced with the increase of forecasting time. It is the inherent disadvantages of the time series forecasting model-the forecasting ability to extrapolate is limited, the longer the forecasting time, the lower the prediction accuracy. Further studies are needed to develop synthetic approaches combining various types of models to improve the ability of forecasting the new admission inpatients from different data.

From a clinical perspective, our research shows that it is benefit to monitor the change trend of admission inpatients by adding time series model to the hospital information system. When the predicted new admission inpatients are increasing, hospital managers can open more preparation beds or let doctors reduce the admissions. From a methodology perspective, our research shows that the time series model can be applied to study the development trend of admission inpatients. NARNN model was implemented based on the neural network time series tool of MATLAB which provided a graphical environment to make the design process of model easy. Although many researches have indicated hybrid models could improve the forecasting performance, our results do not support this point. Understanding how and which models could be implemented in which data requires hospital managers prudent choice.

## Conclusions

In summary, the SARIMA-NARNN model for forecasting did not always provide better estimates than the single NARNN model. Our results show that combined models do not necessarily outperform the individual constituents. Therefore, it is worth attempting to explore different reliable models with high degree of accuracy for forecasting the number of new admission inpatients using different data.

## Additional files

Additional file 1: Original data. The table showed the original data including the number of daily (1/4/2016–2/10/2016) and monthly (1/2010–10/2016) new admission inpatients. (XLS 33 kb)

Additional file 2: Predicted values. The table showed the predicted monthly and daily new admission inpatients from three models. (XLS 54 kb)

## Abbreviations

ARIMA: Autoregressive Integrated Moving Average
MAE: Mean Absolute Error
NARNN: Nonlinear Autoregressive Neural Network
RMSE: Root Mean Square Error
SARIMA: Seasonal Autoregressive Integrated Moving Average
MAPE: Mean Absolute Percentage Error
ANN: Artificial Neural Network
ACF: Autocorrelation Function
PACF: Partial Autocorrelation Function
ADF: Augmented Dickey-Fuller
PP: Phillips and Perron
AIC: Akaike Information Criterion
SBC: Schwarz Bayesian Criterion
MOS: Monthly Original Time Series
DOS: Daily Original Time Series
MRS: Monthly Residual Series
DRS: Daily Residual Series
